# 

**Published:** 1872-08

**Authors:** 


### BOOK NOTICES.

"THE Life that Now Is," being sermons by ROBERT COLLYER, author of "Nature and Life," published by Lee & Shepard, Boston, 351 pp. 12mo, with an elegant steel engraving of the distinguished author. There are sixteen sermons, all treated with practical ability: The Thorn in the Flesh, How Enoch Walked with God, Holiness and Happiness, Marriage, Patience, The Soldier's Grave, Old Age, etc.

A baker's dozen of original humorous dialogues, thirteen in number, by GEORGE M. BAKER, author of "Amateur Dramas," etc.

"The Witness," a New York daily penny paper, has met with a deserved success, which must be gratifying in the highest degree to all good citizens. It began less than a year ago, up an old pair of ricketty stairs, and an eight by ten counting-room. Now it is on the first floor of a handsome, beautiful white building, five stories high, adjoining the office of the "New York Tribune," and there is a busy bustle about it, and a cheeriness that augurs success. It has in its short life grown some fifty per cent., and its long columns of advertisements, from some of the best houses in New York, are substantial evidences of its thrift. A lady on Murray Hill said the other day to her husband: "Be sure to bring the 'Evening Witness.' I think it is the best evening paper of all."

And it is among the best evening papers; for it is the cheapest for its size, and gathers up the most important news of the day since the morning papers came out.

It is one of the best political papers, because it gives impartial extracts from both sides.

It is one of the best family papers, because it is not loaded down with indecent narrations, with profane caricatures of what is passing, and trashy items of news, and low slang.

It is one of the best of religious newspapers, because it gives accounts of church doings in all denominations, and is not forever and ever snapping and snarling at this church, that church, and the other church, not its own. In fact, we don't know what church it does represent, unless it is the great brotherhood of Christians of every name and kindred, whose end and aim is the world's deliverance from want, and sorrow, and sin.

It is one of the best of daily papers, because our daughters can read any number of it without a blush; because our servants can read it without being angered, and without being initiated into the knowledge of all the wrong-doing in the city. Such a paper ought to be sustained by the advertising patronage of our Christian manufacturers, merchants, and bankers, and thus let the world see who are its backers; it is now a morning paper also.

